# Astrocyte-derived exosomes protect hippocampal neurons after traumatic brain injury by suppressing mitochondrial oxidative stress and apoptosis

**DOI:** 10.18632/aging.203508

**Published:** 2021-09-13

**Authors:** Wenqian Zhang, Jun Hong, Hanwen Zhang, Wencheng Zheng, Ying Yang

**Affiliations:** 1 Department of Neurosurgery, Tangshan Gongren Hospital, Tangshan, Hebei 063000, China; 2 Hebei Institute of Head Trauma, Tangshan Gongren Hospital, Tangshan, Hebei 063000, China; 3 Graduate University, North China University of Science and Technology, Tangshan, Hebei 063000, China; 4 Department of Cardiology, Tangshan Gongren Hospital, Tangshan, Hebei 063000, China; 5 Department of Endocrinology, Tangshan Gongren Hospital, Tangshan, Hebei 063000, China

**Keywords:** exosomes, astrocytes, traumatic brain injury, oxidative stress, apoptosis

## Abstract

In this study, we investigated the mechanisms through which astrocyte-derived exosomes (AS-Exos) alleviate traumatic brain injury (TBI)-induced neuronal defects in TBI model rats and mice. Treatment with AS-Exos alleviated neurobehavioral deficits, cognitive impairment, and brain edema in TBI rats. AS-Exos also significantly reduced neuronal cell loss and atrophy in the TBI rats. AS-Exos significantly reduced oxidative stress and mitochondrial H_2_O_2_ levels by increasing the activity of antioxidant enzymes such as superoxide dismutase (SOD) and catalase (CAT) in the hippocampal neurons of TBI rats. TUNEL-staining assays showed that AS-Exos significantly reduced TBI-induced neuronal apoptosis. Mechanistically, AS-Exos ameliorated oxidative stress by activating Nrf2/HO-1 signaling in the hippocampus of TBI rats. In addition, the neuroprotective effects of AS-Exos were abrogated in brain-specific Nrf2-knockout mice subjected to TBI. These findings demonstrate that AS-Exos protects against TBI-induced oxidative stress and neuronal apoptosis by activating Nrf2 signaling in both rat and mouse models.

## INTRODUCTION

Traumatic brain injury (TBI) is related to high morbidity and mortality, and nearly 50 million TBI cases are reported worldwide each year [1, 2]. In China, the average mortality rate for TBI patients is about 13 cases per 100,000 individuals [3]. TBI is classified as primary and secondary cerebral injuries based on the pathophysiological processes involved. Primary TBI is caused by direct mechanical force, whereas, secondary TBI involves pathophysiological processes such as oxidative stress, inflammation response, autophagy, neuronal death, and others [4–6]. Oxidative stress is caused by redox imbalance between reactive oxygen species (ROS) and the antioxidative defense systems in cells and tissues [7]. Mitochondrion is the principal organelle that generates cellular ROS as byproducts of oxidative phosphorylation (OXPHOS), which involves reduction of molecular oxygen via the electron transport chain (ETC) to generate energy-rich ATP molecules [8]. Mitochondrial dysfunction in neuronal cells results in generation of excessive ROS, which causes oxidative damage [9] and programmed neuronal cell death or apoptosis [10]. Current therapeutic interventions for patients with TBI are limited and include drugs that improve nervous functions by reducing oxidative stress and alleviating mitochondrial dysfunction [11].

Several studies have shown neuroprotective effects of brain-targeted drug carriers such as exosomes, which diminish ROS levels by delivering antioxidants to the neurons [12–15]. Exosomes are nano-sized, membrane bound vesicles with a diameter of 50–100 nm that act as key mediators of intercellular communication, traverse the blood–brain barrier (BBB), and carry various molecules such as DNA, RNAs and proteins [16, 17]. The intercellular trafficking of small molecules via exosomes is a promising therapeutic option for various neurological disorders such as Parkinson disease (PD), Alzheimer’s disease (AD), and intracerebral hemorrhage (ICH) [18–20]. Exosomes are released by several cell types including astrocytes [21]. Astrocytes are the most abundant type of glial cells in the central nervous system (CNS) that maintain pH and ion homeostasis, provide metabolic support to the CNS, and promote synaptic formation and remodeling [22, 23]. Astrocytes also play a significant role in maintaining redox balance and decreasing oxidative stress in the brain under normal physiological and pathological conditions [24]. Astrocyte-derived exosomes (AS-Exos) are potent inhibitors of oxidative stress, which is significantly associated with neuronal apoptosis [25, 26]. However, the neuroprotective effects of AS-Exos in TBI are not well known. Therefore, in the present study, we aimed to investigate the beneficial neuroprotective action of AS-Exos in response to TBI and the potential mechanisms using rat and mouse models of TBI.

## RESULTS

### Basic characterization of primary AS-derived exosomes

**Figure 1:**
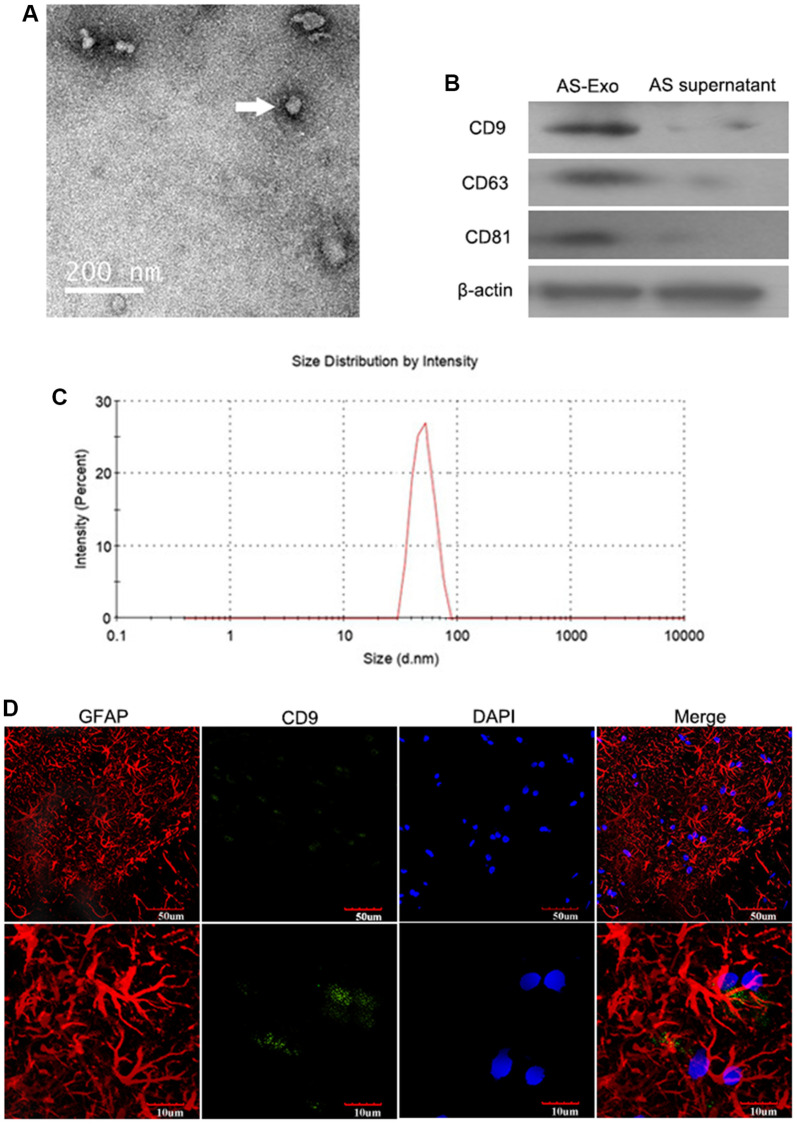
**Characterization of AS-Exosomes.** (**A**) Representative transmission electron microscopy (TEM) image show exosomes generated by primary astrocytes. (**B**) Western blot analysis shows expression levels of exosomal biomarkers such as CD9, CD63, CD81, HSP90B1, and GM130 in astrocyte-derived exosomes (AS-Exo) and astrocyte supernatant. (**C**) Particle size analysis of AS-Exo. (**D**) The identification of astrocytes and AS-Exo.

TEM images of AS-Exos are illustrated in Figure 1A. Western blot showed that exosome-specific markers such as CD9, CD63, and CD81 were highly expressed by the AS-Exos (Figure 1B). Particle size analysis showed that the diameter of the AS-Exos ranged between 30 and 100 nm (Figure 1C). The authentication report of astrocyte was shown in Figure 1D.

### AS-Exos treatment ameliorates neurological dysfunction and cognitive deficits in the TBI model rats

**Figure 2:**
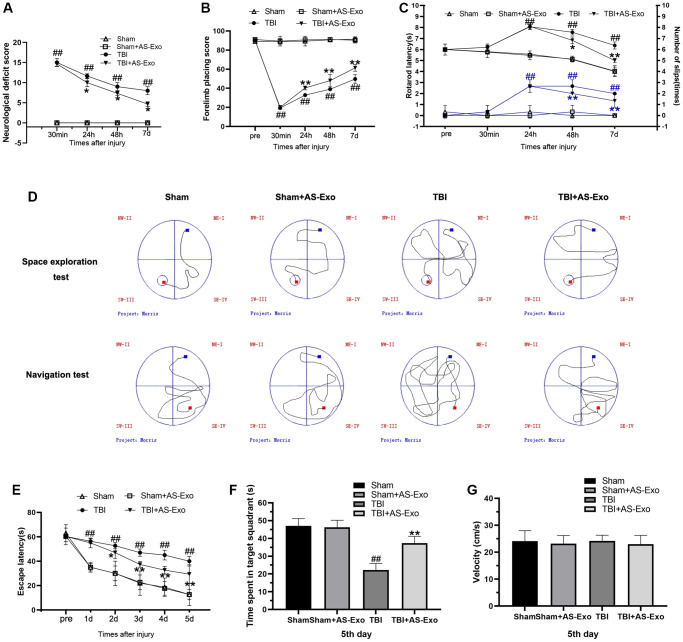
**AS-Exos ameliorate TBI-induced neurobehavioral and cognitive deficits in rats.** (**A**–**G**) Neurological function test results of TBI, TBI+AS-Exo, Sham, and Sham+AS-Exo groups of rats including (**A**) Modified neurological severity score (mNSS), and results of (**B**) Forelimb placement, (**C**) Rotating rod, and (**D**–**G**) MWM tests. All data are represented as means ± standard error (*n* = 8 per group). Statistical significance was determined using one-way ANOVA followed by post-hoc Bonferroni correction. ^#^*P* < 0.05 or ^##^*P* < 0.01 vs. the Sham group; ^*^P < 0.05 or ^**^P < 0.01 vs. the TBI group.

Neurological function of the rats was evaluated by mNSS, forelimb placement test and rotarod test. TBI+AS-Exo group rats showed lower mNSS values compared to the TBS group rats at 24 h, 48 h, and 7 d, but, mNSS values of Sham+AS-Exo group rats were similar to Sham rats (*P* > 0.05) (Figure 2A). Forelimb placement test results showed severe forelimb placing deficits in the TBI group rats at 30 min, 24 h, 48 h, and 7 days compared to the sham-operated rats (*P* < 0.01; Figure 2B). However, treatment with AS-Exos significantly improved forelimb functions at 24 h, 48 h, and 7 days after TBI (*P* < 0.01; Figure 2B). TBI rats demonstrated shorter rotarod latencies were shorter compared with those in Sham group (*P* < 0.01), but, administration with AS-Exos significantly increased rotarod latencies over time after TBI (*P* < 0.01) (Figure 2C). Rotarod latencies of the Sham and Sham+AS-Exo group rats were similar (*P* > 0.05; Figure 2C). MWM trial showed significant increase in escape latency of the TBI group rats over the course of the navigation test compared to sham-treated rats (*P* < 0.01; Figure 2D), but remarkable improvements were observed in the TBI+AS-Exo group rats (*P* < 0.05) (Figure 2E). During space exploration tests, target quadrant residence time was significantly shorter for the TBI group rats relative to the Sham-operated rats (*P* < 0.01), but TBI+AS-Exo group rats showed significant improvements compared to the TBI group rats; time spent in the target quadrant was similar for both Sham and Sham+AS-Exo group rats (*P* > 0.05; Figure 2F). As shown in Figure 2G, there is no significant difference in swimming speed was observed among groups (*P* > 0.05). Overall, these results suggested that treatment with AS-Exos significantly improved neurological functions and cognition in the TBI model rats.

### AS-Exos reduced brain edema and pathology in the TBI model rats

**Figure 3:**
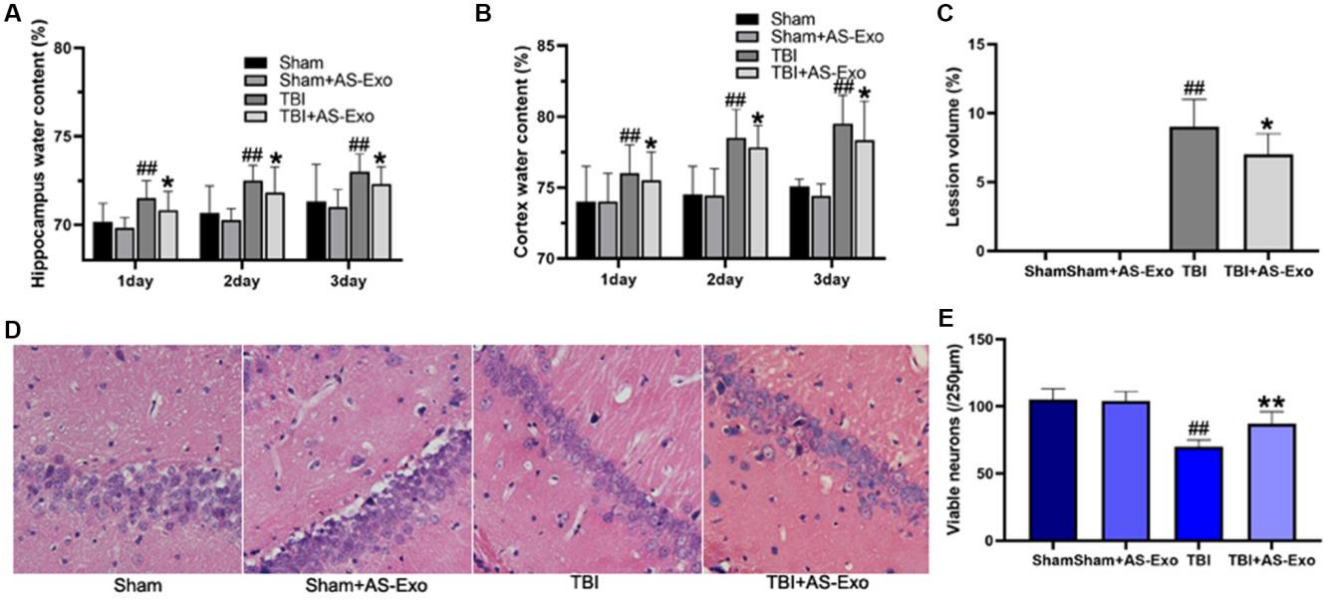
**AS-Exos reduce TBI-induced brain edema, lesion volume and neuronal damage.** (**A**) Hippocampus water content, (**B**) Cortex water content, and (**C**) Lesion volume in the hippocampal tissues of TBI, TBI+AS-Exo, Sham, and Sham+AS-Exo groups of rats. (**D**) Representative H&E stained images of hippocampal tissue sections from TBI, TBI+AS-Exo, Sham, and Sham+AS-Exo groups of rats. (**E**) Quantitative analysis shows the number of viable neurons per 250 μm length of the CA1 pyramidal cell layer in the H&E stained hippocampal tissue section from the four groups of rats. All data are represented as means ± SEM (*n* = 8 per group). Statistical significance was determined using one-way ANOVA followed by post-hoc Bonferroni correction. ^#^*P* < 0.05 or ^##^*P* < 0.01 vs. Sham group; ^*^P < 0.05 or ^**^P < 0.01 vs. TBI group.

We then measured brain water content and lesion volume on three consecutive days after TBI to determine the effects of AS-Exo on the brain pathology of TBI model rats. Water content of the cortex and hippocampus was dramatically elevated on 3 consecutive days following TBI compared to those in Sham group (*P* < 0.01), but AS-Exo treatment significantly reduced cortex and hippocampus edema after TBI on three consecutive days (all *P* < 0.05; Figure 3A–3B). Moreover, AS-Exo reduced brain lesion volume on the third day post-TBI relative to the TBI rats (*P* < 0.05, Figure 3C).

Next, we performed H&E staining to determine TBI-induced brain pathology and the effects of AS-Exo treatment. H&E staining of hippocampal tissues from the Sham and Sham+AS-Exo rat groups showed normal tissue morphology, including intact nuclei and abundant cytoplasm (Figure 3D). Hippocampal neurons in the TBI group showed neuronal atrophy, neuronal loss, nuclear pyknosis, and uneven cytoplasmic staining, but treatment with AS-Exos significantly reduced neuronal cell loss (Figure 3E) and atrophy (Figure 3D) in the TBI model rats *P* < 0.05.

### AS-Exos ameliorate TBI-induced oxidative stress in the rat hippocampus

**Figure 4:**
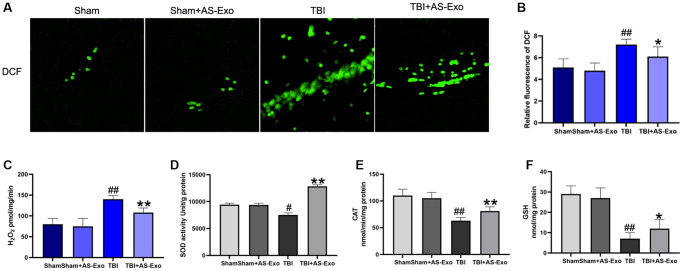
**AS-Exos alleviates TBI-induced oxidative stress in the rat hippocampal tissues.** (**A**) Fluorescence images show DCF-stained hippocampal tissues from TBI, TBI+AS-Exo, Sham, and Sham+AS-Exo groups of rats. (**B**) Bar graphs illustrate relative DCF fluorescence intensity in the hippocampal tissues from the four groups of rats. (**C**) Amplex red hydrogen peroxide/peroxidase assay results show release of mitochondrial H_2_O_2_ in the four groups. (**D**) SOD activity, (**E**) CAT activity and (**F**) Reduced GSH levels in the hippocampal tissues isolated from TBI+AS-Exo, Sham, and Sham+AS-Exo groups of rats (48 h after TBI). All data are represented as means ± SEM (*n* = 5 per group). Statistical significance was determined using one-way ANOVA followed by post-hoc Bonferroni correction. ^#^*P* < 0.05 or ^##^*P* < 0.01 vs. Sham group; ^*^P < 0.05 or ^**^P < 0.01 vs. TBI group.

We then examined ROS and mitochondrial H_2_O_2_ levels in the hippocampal tissues of rats belonging to different experimental groups. Cellular ROS and mitochondrial H_2_O_2_ levels were significantly increased in the hippocampal tissues of TBI rats after 48 h (Figure 4A–4C). This suggested that TBI increased oxidative stress in the hippocampal tissues. However, treatment with AS-Exo significantly reduced cellular ROS and mitochondrial H_2_O_2_ levels in the hippocampal tissues of TBI rats (*P* < 0.05); ROS and mitochondrial H_2_O_2_ levels were comparable in the hippocampal tissues of Sham and Sham+AS-Exo group rats (*P* > 0.05; Figure 4A–4C).

Next, we analyzed superoxide dismutase (SOD) and catalase (CAT) enzyme activities, as well as reduced GSH levels in the hippocampal neurons of all groups of rats to determine the effects of TBI and AS-Exos on antioxidant capacity. We observed significant reduction in the SOD, CAT and reduced GSH levels of the hippocampal tissues of TBI model rats after 48 h, but administration of AS-Exo significantly reversed these effects in the TBI model rats (Figure 4D–4F). However, SOD and catalase activity and reduced GSH levels were similar in the hippocampus tissues of Sham and Sham+AS-Exo group rats (*P* > 0.05; Figure 4D–4F).

### AS-Exo upregulates Nrf2 signaling pathway in hippocampal neurons of TBI model rats

**Figure 5:**
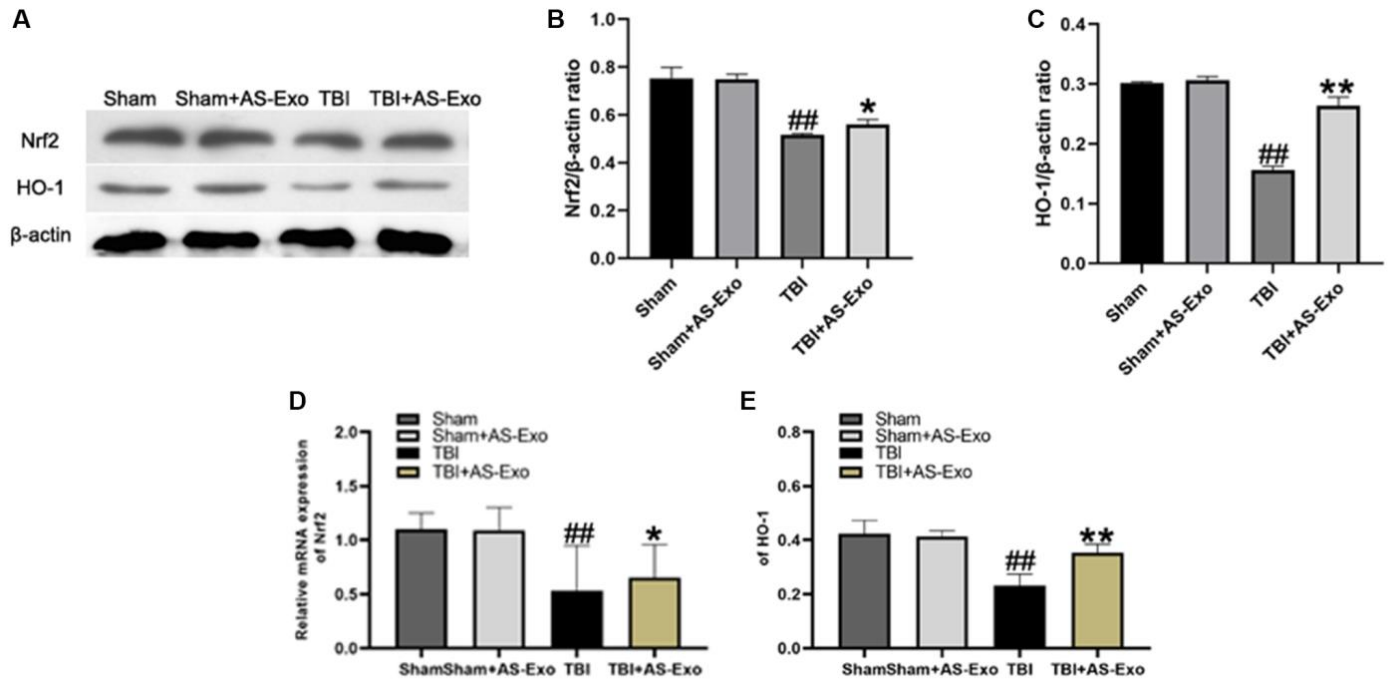
**AS-Exos activate Nrf2/HO-1 pathway in the hippocampal neurons of TBI-induced rat brains.** (**A**) Western blot analysis shows Nrf2 and HO-1 protein expression levels in the hippocampus of TBI+AS-Exo, Sham, and Sham+AS-Exo groups of rats at 48 h after TBI or sham surgery. (**B**–**E**) Bar graphs show the relative expression levels of (**B**) Nrf2 and (**C**) HO-1 proteins, (**D**) Nrf2 and (**E**) HO-1 mRNAs in the hippocampus tissues from the four groups of rats at 48 h after TBI or sham surgery. All data are represented as means ± SEM (*n* = 5 per group). Statistical significance was determined using one-way ANOVA followed by post-hoc Bonferroni correction. ^#^*P* < 0.05 or ^##^*P* < 0.01 vs. Sham group; ^*^P < 0.05 or ^**^P < 0.01 vs. TBI group.

Next, we analyzed the status of the Nrf2 signaling pathway in hippocampal tissues in different groups. Nrf2 and HO-1 mRNA and protein levels were significantly reduced in the hippocampal tissues of TBI group rats compared to the Sham group rats, (*P* < 0.01), but were significantly higher in the TBI+AS-Exo group compared to the TBI group (*P* < 0.05; Figure 5). Nrf2 and HO-1 protein and mRNA levels were similar in Sham and Sham+AS-Exo group rats (*P* > 0.05; Figure 5). These results suggested that AS-Exos activated Nrf2 signaling pathway in the hippocampal neurons of TBI model rats.

### AS-Exo reduces TBI-induced neuronal apoptosis

**Figure 6:**
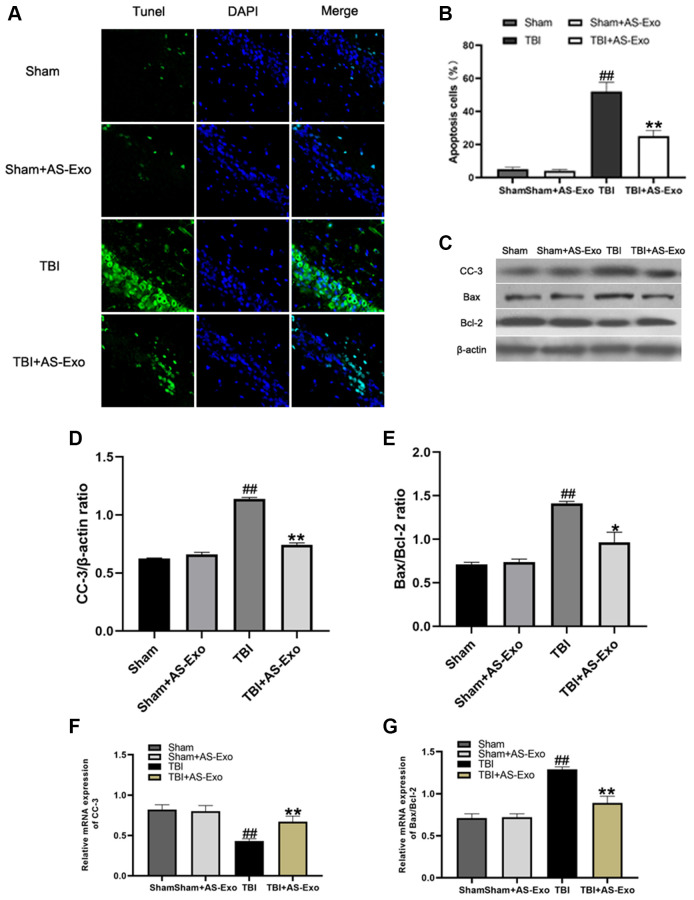
**AS-Exos reduce TBI-induced neuronal apoptosis.** (**A**) Representative confocal images (scale bar, 50 μm) show neuronal apoptosis in hippocampus tissues from TBI+AS-Exo, Sham, and Sham+AS-Exo groups of rats at 48 h after TBI or sham surgery based on TUNEL (green) and DAPI (blue) staining. (**B**) Bar graph shows the relative percentage of apoptotic neuronal cells in the hippocampus tissues based on TUNEL staining from the four groups of rats at 48 h after TBI or sham surgery. (**C**) Western blot analysis shows expression levels of CC-3 Bax, and Bcl-2 proteins in the hippocampus tissues from TBI+AS-Exo, Sham, and Sham+AS-Exo groups of rats at 48 h following TBI or Sham surgery. (**D**–**E**) Bar graphs illustrate densitometry analyses of (**D**) CC-3 and (**E**) Bax/Bcl-2 ratio. The protein bands were normalized to β-actin. (**F**–**G**) Bar graphs illustrate qRT-PCR results for the expression levels of (**F**) CC-3 and (**G**) Bax/Bcl-2 mRNAs relative to β-actin in hippocampus tissues from TBI+AS-Exo, Sham, and Sham+AS-Exo groups of rats at 48 h after TBI or sham surgery. All data are represented as means ± SEM (*n* = 5 per group). Statistical significance was determined using one-way ANOVA followed by post-hoc Bonferroni correction. ^#^*P* < 0.05 or ^##^*P* < 0.01 vs. Sham group; ^*^P < 0.05 or ^**^P < 0.01 vs. TBI group.

We then assessed if AS-Exos reduced TBI-induced neuronal apoptosis using terminal deoxynucleotidyl transferase dUTP nick end labeling (TUNEL) assay. The numbers of TUNEL-positive (apoptotic) hippocampal neurons were significantly higher in the TBI group compared to the Sham group (*P* < 0.01; Figure 6A). However, the proportion of TUNEL-positive hippocampal neurons was significantly reduced in the TBI+AS-Exo group compared to the TBI group (*P* < 0.01; Figure 6B).

We then analyzed protein and mRNA levels of CC-3, Bax and Bcl-2 in the hippocampal tissues of various rat groups. Western Blot and qRT-PCR analyses showed that Bax to Bcl-2 ratio and CC-3 levels were significantly increased after TBI, while decreased after AS-Exo treatment (*P* < 0.01; Figure 6C–6G), suggesting that AS-Exos ameliorated neuronal apoptosis in hippocampus of TBI rats.

### AS-Exos reduce oxidative stress by activating Nrf2 signaling pathway

**Figure 7:**
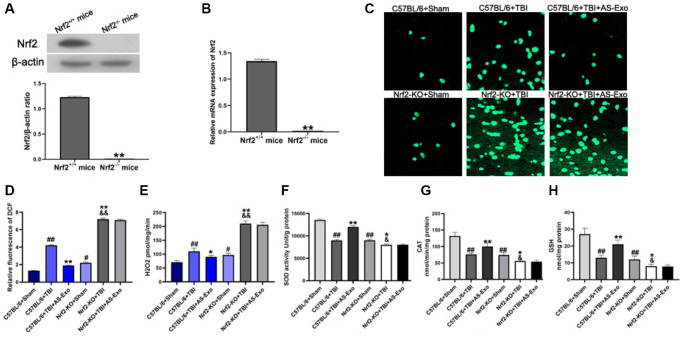
**AS-Exos reduce oxidative stress in hippocampal neurons of TBI-induced murine brains by activating Nrf2 signaling pathway.** (**A**) Western blot analysis and (**B**) PCR analysis confirms Nrf2 knockdown in Nrf^−/−^ mice. ^**^P < 0.01 vs. Nrf2^+/+^ mice. (**C**) Representative confocal images show DCF fluorescence in the hippocampus tissues from Nrf-KO+Sham, Nrf^−^-KO+TBI, Nrf^+/+^+Sham, and Nrf^+/+^+TBI mice injected with or without AS-Exos. (**D**) Bar graphs illustrate relative DCF fluorescence in the hippocampus tissues from the mice groups. (**E**) Amplex red hydrogen peroxide/peroxidase assay results show the release of mitochondrial H_2_O_2._ Mitochondria were isolated from the hippocampus tissues of the mice groups. (**F**) SOD activity, (**G**) CAT activity and (**H**) Reduced GSH levels in the hippocampus tissues from the mice groups. Data are represented as means ± SD (*n* = 5 per group). Statistical significance was determined using one-way ANOVA followed by post-hoc Bonferroni correction. ^#^*P* < 0.05 or ^##^*P* < 0.01 vs. C57BL/6+Sham; ^*^P < 0.05 or ^**^P < 0.01 vs. and Nrf^+/+^+TBI group; ^&^*P* < 0.05 or ^&&^*P* < 0.01 vs. Nrf2-KO+Sham group.

Next, we used brain-specific Nrf2 knockout mice (C57BL/6 background) to explore the potential mechanisms underlying the neuroprotective effects of AS-Exos. First, we confirmed Nrf2 knockdown in the hippocampus of brain-specific Nrf2 knockout mice by western blotting and PCR analysis (Figure 7A–7B). Then we assessed the status of Nrf2/HO-1 pathway activation and antioxidant response pathway. The hippocampal tissues of TBI+Nrf2^+/+^ mice showed significantly higher ROS and H_2_O_2_ levels as well as decreased activity of SOD and CAT as well as reduced GSH levels compared with the Sham+Nrf2^+/+^ mice (*P* < 0.01; Figure 7C–7H). However, AS-Exos decreased ROS and H_2_O_2_ levels, and increased SOD and CAT activities, and reduced GSH levels in TBI+AS-Exo+Nrf2^+/+^ mice (Figure 7C–7H). Nrf2-KO+Sham mice showed increased oxidative stress compared to the Sham+Nrf2^+/+^ mice (Figure 7C–7H). Furthermore, Nrf2-KO+TBI mice displayed significantly higher levels of ROS and H_2_O_2_ as well as reduced SOD and CAT activities and reduced GSH levels than other mouse groups, but these effects were not reversed in the Nrf2-KO+TBI+AS-Exo group mice (Figure 7C–7H). This demonstrated that AS-Exos reduced oxidative stress in hippocampal neurons through the Nrf2 signaling pathway.

### AS-Exos reduce TBI-induced apoptosis by activating Nrf2 signaling pathway

**Figure 8:**
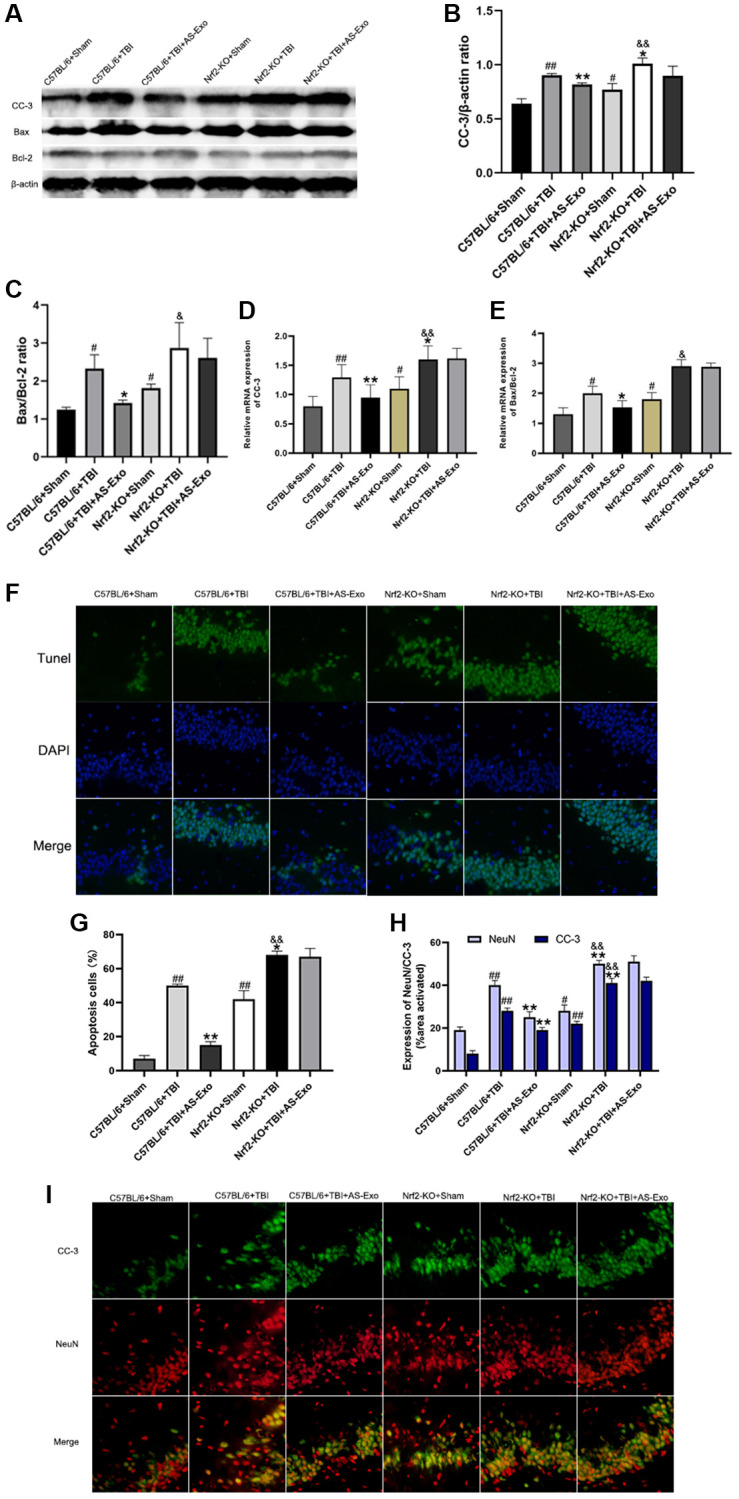
**AS-Exos decrease TBI-induced neuronal apoptosis by activating Nrf2.** (**A**) Western blot analysis shows expression levels of CC-3 and ratio of Bax/Bcl-2 proteins in the hippocampus of mice groups at 48 h following TBI or Sham surgery. (**B**–**C**) Bar graphs show (**B**) CC-3 protein expression levels and (**C**) ratio of Bax/Bcl-2 proteins normalized to β-actin. (**D**–**E**) Bar graphs show the (**D**) CC-3 mRNA expression levels and (**E**) ratio of Bax/Bcl-2 mRNAs relative to β-actin mRNA levels in the hippocampus tissues of the mice groups. (**F**) Representative confocal images show TUNEL (green) and DAPI (blue) staining of hippocampus tissue sections from the mice groups. (**G**) Bar graph shows the relative percentage of apoptotic neuronal cells in the hippocampus tissues from various mice groups. (**H**) MATLAB software analysis shows the staining intensities for NeuN and CC-3 in the hippocampus tissues of various mice groups. (**I**) Representative images show double immunofluorescence staining of NeuN (red) and CC-3 (green) in the hippocampus tissues of various mice groups. (Scale bar, 100 μm). Data are represented as means ± SD (*n* = 5 per group). ^#^*P* < 0.05 or ^##^*P* < 0.01 vs. C57BL/6+Sham; ^*^P < 0.05 or ^**^P < 0.01 vs. C57BL/6+TBI group; ^&^*P* < 0.05 or ^&&^*P* < 0.01 vs. Nrf2-KO+Sham group.

Lastly, we explored whether AS-Exos reduced neuronal apoptosis after TBI by activating Nrf2. QRT-PCR and western blot showed that CC-3 expression and the Bax to Bcl-2 ratio was significantly higher in the TBI+Nrf2^+/+^ mice compared to the Sham+Nrf2^+/+^ mice (Figure 8A–8G). However, reduced CC-3 and the Bax to Bcl-2 ratio was observed in the AS-Exo+TBI+Nrf2^+/+^ mice versus the TBI+Nrf2^+/+^ mice (both *P* < 0.05). The expression of CC-3 and Bax to Bcl-2 ratio was elevated in the Nrf2-KO+Sham mice compared to the Sham+Nrf2^+/+^ mice (Figure 8A–8G). Moreover, as compared to Nrf2-KO+Sham group, Bax to Bcl-2 ratio and CC-3 was significantly higher in the Nrf2-KO+TBI group (both *P* < 0.05; Figure 8A–8G), but these results were not reversed by addition of AS-Exos (*P* > 0.05; Figure 8A–8G).

We also performed double immunofluorescence staining of murine hippocampal tissues with antibodies against CC-3 and neuronal nuclei (NeuN). TBI+Nrf2^+/+^ mice showed increased numbers of apoptotic neurons and increased expression of CC-3 compared to the Sham+Nrf2^+/+^ mice, but these effects were reversed by AS-Exos (Figure 8H–8I). Nrf2-KO+Sham mice showed significantly higher numbers of apoptotic neurons and increased CC-3 expression levels compared to the Sham+Nrf2^+/+^ mice (Figure 8H–8I). Furthermore, Nrf2-KO+TBI mice showed higher CC-3 expression and increased numbers of apoptotic neurons compared to Nrf2-KO+Sham mice, but these effects were not reversed by AS-Exos (Figure 8H–8I). These results suggested that AS-Exos inhibited TBI-induced neuronal apoptosis by activating Nrf2.

### DISCUSSION

TBI-induced neurological dysfunction and cognitive deficits significantly affect prognosis of patients with TBI [27]. Our study showed that AS-Exos significantly alleviated TBI-induced short-term and long-term memory/learning defects. Previous studies have shown that increased blood-brain barrier permeability in response to TBI promoted the release of cytotoxic mediators and ROS [28]. TBI also causes intracranial pressure by increasing brain volume and lowering brain oxygenation levels [28]. Currently, TBI-induced brain edema is treated symptomatically and drugs that reduce brain edema are not available for standard therapy [29]. Our results demonstrated that AS-Exos significantly reduced water content and lesion volumes in both the cortex and hippocampus of TBI model rats. Moreover, AS-Exo treatment attenuated structural alterations and neuronal death in the hippocampus of TBI model rats. Our main experimental findings are supported by Chen et al. who reported that AS-Exos induced neuronal recovery after TBI [30]. Therefore, AS-Exos are promising therapeutic avenues for a wide variety of brain-related diseases including TBI [31].

Astrocytes are highly enriched in the brain, and play a critical role in information processing, neuronal development, and neurological function maintenance [32]. Many studies have confirmed that astrocytes communicate with neurons through extracellular vesicles (EVs) [33, 34]. Our study also demonstrated that AS-Exos reduced TBI-induced apoptosis of hippocampal neurons by decreasing H_2_O_2_ and ROS levels, as well as increasing SOD and CAT activities and reduced GSH levels. Oxidative stress is caused by imbalance between cellular ROS production and antioxidant defense mechanisms [35]. Mitochondria are the main source of cellular ROS and oxidative stress [36]. Guitart et al. reported that prion protein derived from AS-Exos improved neuronal survival under hypoxic and ischemic conditions [25]. AS-Exos also decreased apoptosis and oxidative stress of hippocampal neurons under oxygen and glucose deprivation conditions [37]. Du et al. showed that AS-Exos protected neonatal rats from hypoxic-ischemic brain damage by inhibiting BNIP-2 expression via miR-17-5p [37]. Therefore, we postulated that AS-Exos protected hippocampal neurons against TBI-induced damage through complex and diverse antioxidant mechanisms.

Oxidative stress and neuronal apoptosis correlate with TBI-induced neurological impairment and cognitive dysfunction [38]. Mitochondrial dysfunction is associated with the intrinsic pathway of apoptosis in neurons [35]. Therefore, improving mitochondrial function is an important focus of treating patients with TBI [39]. In the present study, we found that TBI induced a cascade of effector caspases and altered the Bax/Bcl-2 ratio, but these effects were abrogated by AS-Exos. Under normal physiological conditions, astrocyte-derived EVs exhibit neuroprotective properties [34, 40]. AS-EV-derived neuronal survival factor protected neuronal cells from excitotoxicity under OGD and glutamate-stress [41, 42]. Therefore, AS-Exos is a potential neuroprotective treatment for patients with TBI.

AS-Exos exert anti-oxidative and anti-apoptotic effects through multiple mechanisms [25, 37]. The activation of Nrf2 in astrocytes protected proximal neurons against oxidative stress-related injury and apoptosis [43]. Nrf2 pathway was associated with protein and cellular redox homeostasis as well as intracellular metabolism [44]. Wang et al. showed that mesenchymal stem cell-derived exosomes repaired oxidative stress-induced skin injury by reducing ROS levels, DNA damage, and mitochondrial changes through NRF2 signaling pathway [45]. Exosomes derived from bovine granulosa cells and bovine milk exosomes protected against cellular oxidative stress by elevating antioxidant capacity through activation of Nrf2 pathway [46, 47]. This study showed that AS-Exos decreased ROS production and neuronal apoptosis by enhancing antioxidant mechanisms through activation of Nrf2. These findings demonstrate immense potential for the use of AS-Exos in neuroprotective therapy for TBI.

Our study has few drawbacks that need to be addressed in future investigations. Firstly, brain-specific conditional Nrf2 knockout mice should be used in future studies to demonstrate that Nrf2 suppresses mitochondrial oxidative stress and apoptosis after TBI. Secondly, whole transcriptome sequencing of AS-Exos is necessary to identify and confirm neuroprotective components. Thirdly, the effects of Nrf2 on neuroinflammation and blood brain barrier disruption require further in-depth study.

**Figure 9:**
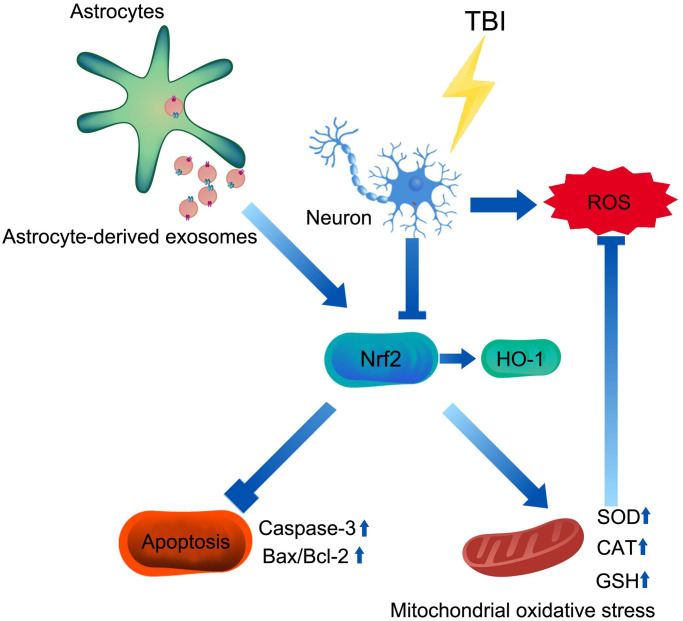
**Mechanism of AS-Exos alleviating TBI-induced neuropathology.** AS-Exosomes attenuate neuronal injury after TBI by inhibiting mitochondrial oxidative stress and apoptosis via activation of the Nrf2 pathway.

In conclusion, our study suggested that AS-Exos protected against TBI-induced neuronal oxidative stress and apoptosis through activation of Nrf2 signaling pathway (Figure 9).

## MATERIALS AND METHODS

### Primary astrocyte culture

Primary rat astrocytes were isolated by dissecting cerebral cortices of 1–2 newborn Sprague-Dawley (SD) rats under the microscope and digesting them with 0.25% trypsin as previously described [48]. The primary cells were then grown in Eagle Minimum Essential Medium (EMEM) containing 10% equine serum, 21 mM glucose, 10% fetal bovine serum, and epidermal growth factor (10 ng/mL, EGF) at a cell density of 1–1.5 hemispheres per well in a humidified chamber at 37°C and 5% CO_2_.

### Isolation of astrocyte exosomes (AS-Exos)

AS-Exos were isolated from the culture medium of primary rat astrocytes by differential centrifugation as previously described [49]. In brief, 70–90% confluent primary rat astrocytes were grown in medium with exosome-depleted FBS for 72 h. Subsequently, the cell suspension was centrifuged at 2,000 g for 30 min. The pelleted cells and cell debris were discarded. The supernatant was then centrifuged at 10,000 × g for 40 min and the pelleted microvesicles were discarded. Then, the AS-Exo pellet was obtained by centrifuging the supernatant at 100000 × g for 2 h at 4°C. The AS-Exo pellet was resuspended in PBS and its morphology was analyzed by electron microscopy (EM, Hitachi H-7600, Hitachi, Tokyo, Japan). The particle size of AS-Exo was confirmed by Nanosizer^™^ technology (Malvern Instruments, Malvern, Worcestershire, UK). Western blot was carried out to detect expression levels of exosomal surface markers, namely, CD9, CD63, and CD81.

### Establishment of TBI model rats and mice

Ethics approval was granted through Ethics Committee of Tangshan Gongren Hospital (GRYY-LL-2020-101). Animal experiments were in accordance with the guidelines of the National Institutes of Health Guide for the Care and Use of Laboratory Animals [50]. Adult male SD rats (weight: 210–260 g) were obtained from the Experimental Animal Center of North China University of Science and Technology and housed under standard conditions (22 ± 1°C, 12-h light/12-h dark, 50 ± 10% humidity) with free access to food and water. The rat TBI model was established based on the method described in a previous study [51]. All the rats (60) were randomly split into four groups (*n* = 15 per group): Sham, Sham+AS-Exo, TBI, and TBI+AS-Exo. The exosome concentration was estimated using Pierce bicinchoninic acid (BCA) Protein Assay Kit (Thermo Scientific, USA) according to the manufacturer’s instructions. We then injected 100 μg AS-Exos via the tail vein, 30 min after the rats underwent TBI. One rat died in the TBI group during the modeling process.

Brain-specific Nrf2 knockout mice (C57BL/6 background) were obtained from Saiye Biotech Limited Company (Guangzhou, China). Western blot and quantitative RT-PCR analyses were performed to distinguish Nrf2 knockout (Nrf2-KO) and Nrf2 wild-type (Nrf2^+/+^) mice. The mice grouping (*n* = 30 per group), TBI modeling, and administration of AS-Exos was performed as described earlier.

### Modified neurological severity score (mNSS)

Neurological functional assessment was performed at 30 min, 24 h, 48 h, and 7 days after TBI. The animals in all groups were assigned a modified neurological severity score (mNSS) [52]. Higher scores denote worse neurological defects.

### Forelimb placement and Rotating rod tests

Forelimb placement and Rotarod performance tests were carried out to evaluate forelimb dysfunction and motor coordination in the rodents. The tests were performed before TBI and 30 min, 24 h, 48 h, and 7 days following TBI. In the forelimb placement test [53], rats were suspended by the holding on their torso and allowed the forelimbs hanging free. The rats were gently pulled upward to relax their muscles before the test. Each side was tested individually by brushing the respective vibrissae on the edge of a tabletop. Normal rats would promptly place the ipsilateral forelimb on the desktop. Each rat was measured 10 times in each forelimb, and the percentage of correct responses was determined.

The rotating rod test was performed as reported previously [54]. In the test, the rod accelerated from 5 rpm to 40 rpm. The speed of the rotating drum was increased gradually and the average time that the rats could hold onto the rotating drum was recorded after the second fall. All data were recorded manually by two independent investigators. Three trials were conducted on each rat per day.

### Morris water maze (MWM) cognitive function tests

As previously described [51, 55], MWM tests were conducted to assess the capacity to learn and remember information on days 1–5 after TBI. The test included two parts: (1) positioning navigation test; (2) space exploration test. The rats were placed individually in a circular MWM apparatus and allowed to a free 5 min swim before conducting the positioning navigation test. A platform was placed in the middle of one of the quadrants. The rats were put into the water in one of the remaining three quadrants. At a indicated time of each training day, rats were picked up to locate in the centre of the hidden platform. If the subject rat boarded the hidden platform within 90 s, it was allowed to stay on the platform and explored for an additional 15 s. Otherwise, the rat would be manually guided to locate the platform and given 15 s to stay. The time was marked as 90 s. The test was performed four trials a day for four days for each mouse.

During the space exploration test on the fifth day, the hidden platform was removed from the tank, and each rat were allowed to swim freely in the pool for 60 seconds. The movement trajectory was drawn and the time spent by the rats in the target quadrant was documented. In order to analyze the performance, the data was record by a video camera which interfaced with a video tracking system (HVS Image Software Ltd., Hampton, UK).

### Brain edema and lesion volume measurements

We measured the brain water content in the animals using the dry-wet method for three days consecutively after TBI or sham operation as described previously [29]. After anesthetization, the rats were killed by decapitation and their brain tissues were immediately removed. The cortex and hippocampus were harvested separately to evaluate their water content for three days after sham operation or TBI. The wet weight (WW) of the brain samples was determined on a precision microbalance. Then, the samples were oven-dried at 100°C for 24 h and the dry weight (DW) was obtained. Water content percentage was calculated as: % brain water = ((WW − DW)/WW) × 100. On the third day after TBI or sham operation, the lesion volume of rats was estimated. After anesthetization, brain tissues were harvested after transcardial perfusion with ice-cold PBS, and fixed by perfusion with 4% paraformaldehyde. Serial coronal brain tissue sections were cut at 0.5-mm intervals and lesion volumes were measured using the Image J software (Image Lab 4.1; National Institutes of Health, USA) [56]. The lesion volume of each section was obtained by multiplying the affected areas by 0.5 mm. The total lesion volume was computed as the sum of infarct areas in the 9 slices taken from the anterior to the posterior limits of the lesion.

### Hematoxylin and eosin (HE) staining

Brain tissues were isolated and obtained 48 h after TBI, embedded in paraffin wax blocks, and cut into 5 μm thick coronal slices. The brain slices were counterstained with hematoxylin solution for 2 min and stained by eosin for 30 seconds. Visualization of histopathological alteration in region of the hippocampus was carried out with an Olympus microscope (Olympus Corporation, Tokyo, Japan; 200× magnification). Number of neurons were counted every 250 μm length per field of vision. Five fields of view were randomly assessed for each rat and the average numbers of neurons per rat were estimated.

### Intracellular ROS measurement

Intracellular ROS was estimated by staining brain samples isolated at 48 h after TBI with a ROS-sensitive fluorescent dye, 2,7-dichlorofluorescein diacetate (DCFH-DA; Sigma-Aldrich, USA). After incubation with 10 μM DCFH-DA for 30 min, brain slices were washed with PBS, and DCF fluorescence was photographed and quantified via laser confocal microscopy (Olympus Corporation, Tokyo, Japan).

### Isolation of brain mitochondria

Purified mitochondria were isolated using the mitochondrial isolation kit (Qiagen, Hilden, Germany) from brain tissue samples harvested at 48 h after TBI. Briefly, hippocampal tissues were homogenized in lysis buffer on ice using a tissue rotor-stator homogenizer for 10 s and centrifuged at 6000 rpm for 10 min at 4°C. The supernatants were transferred into a new tube to collect intact mitochondria. The isolated mitochondria were stored at −80°C until use.

### Estimation of mitochondrial H_2_O_2_ levels

H_2_O_2_ levels were quantified using the amplex red hydrogen peroxide/peroxidase assay kit (Molecular Probes, Monza, Italy) in hippocampal tissues harvested from the animals at 48 h after TBI. Fluorescence intensity was measured using the Fusion Universal Microplate Analyzer (Packard/ PerkinElmer, Milan, Italy) with a 550-nm excitation filter and a 590-nm emission filter. H_2_O_2_ levels were calculated using the standard curve of H_2_O_2_ as previously described [57].

### Antioxidant enzyme activity assays

The antioxidant capacity of the brain tissues was evaluated by assaying activities of SOD and CAT, and the levels of reduced glutathione (GSH). SOD activity was estimated using the Superoxide Dismutase Activity Assay kit (Abcam, Tokyo, Japan) according to the manufacturer’s instructions. The activity of CAT was determined by the Catalase Assay Kit (Cayman Chemical, USA) according to manufacturer’s instructions. Reduced GSH levels were analyzed using the GSH Assay kit (Cayman Chemical, USA) in accordance with the manufacturer’s instructions.

### Quantitative real-time PCR (qRT-PCR)

Total cellular RNA was extracted using the TRIzol^®^ reagent (Invitrogen, Thermo Fisher Scientific, Inc., USA) in accordance with the recommendations of the manufacturer. Complementary DNA (cDNA) synthesis was done by the PrimeScript^™^ RT reagent kit (Cat. No. RR037A; Takara Bio, Inc., Dalian, China) and the synthesis conditions were 37°C for 15 min, 85°C for 5 seconds, and cooling at 4°C. As reported previously [58], TB Green^®^ Premix Ex Taq^™^ II kit (cat. no. RR820A; Takara Bio, Inc., Dalian, China) was used for Quantitative PCR (qPCR). Total reaction volumes equaled 20 ul, with 2 μL template cDNA, 10 μL 2X, 0.8 μL forward primer (10 μM), 0.8 μL reverse primer (10 μM) and 6.4 μL ddH2O per reaction. Thermocycling condition was 37°C for 30 min; 95°C for 1 min; 40 cycles of 18 seconds at 95°C and 60°C for 55 seconds, 72°C for 2.5 min; final hold at 4°C. Each sample was measured three times in each experiment. The sequences of q-PCR primer were as follows:

Cleaved caspase-3 (*CC-3*; forward) 5′-AGCAATAAA TGAATGGGCTGAG-3′, *CC-3* (reverse) 5′-GTATGG AGAAATGGGCTGTAGG-3′; *Bax* (forward) 5′-GTTG CCCTCTTCTACTTTGC-3′, *Bax* (reverse) 5′-ATGGT CACTGTCTGCCATG-3′; *Bcl-2* (forward) 5′-GGTCC TCCAGTGGGTATTT-3′, *Bcl-2* (reverse) 5′-TCCTCC TGAGACTGCCTTAT-3′; *Nrf2* (forward) 5′-GAGACG GCCATGACTGAT-3′, *Nrf2* (reverse) 5′-TGAGGGGA TCGATGAGTA-3′; *HO-1* (forward) 5′-ACAGAAGAG GCTAAGACCGC-3′, *HO-1* (reverse) 5′-GAGCGGTG TCTGGGATGAAC-3′; *β-actin* (forward) 5′-TGACGT GGACATCCGCAAAG-3′, *β-actin* (reverse) 5′-CTGG AAGGTGGACAGCGAGG-3′. *β-actin* mRNA levels were chosen as an internal reference. Relative gene expression was calculated with the 2^−ΔΔCq^ method as previously described [59].

### Western blot

Hippocampus tissues were lysed in radioimmunoprecipitation assay buffer (RIPA, Thermo Fisher Scientific, Inc.). Bicinchoninic Acid Protein Assay Kit (Beyotime, Beijing, China) was used to measure the total protein concentration. Isolated proteins were separated on 12% sodium dodecyl sulfate polyacrylamide (SDS-PAGE) gels. Then, the separated proteins were transferred onto PVDF membranes (Bio-Rad Laboratories, Inc., USA) by electroblotting. After blocked in a nonfat dry milk buffer for 1.5 h at room temperature, the membranes were washed 3 times in PBS containing 0.2%Tween-20. Then, incubation with primary antibodies was accomplished overnight at 4°C: anti-Nrf2 (ab137550; rabbit polyclonal, Abcam), anti-HO-1 (ab13243; rabbit monoclonal, Abcam), anti-Bax (ab32503; rabbit monoclonal, Abcam), anti-Bcl-2 (ab196495; rabbit polyclonal, Abcam), anti-CC-3 (ab49822; rabbit polyclonal, Abcam), anti-CD9 (ab92726; rabbit monoclonal, Abcam), anti-CD63 (sc-5275; mouse polyclonal, Santa Cruz Biotechnology, Inc., USA), anti-CD81 (ab109201; rabbit monoclonal, Abcam), and anti-β-actin (ab8227; rabbit polyclonal, Abcam). The membranes were rinsed and then the secondary antibody (ab216773 or ab216772; Abcam) was incubated at room temperature for 2 h. The blots were developed with the ECL reagent (Bio-Rad Laboratories, Inc., USA). Densitometry estimation of the protein bands was performed using the Image Lab 4.1 software (Image J; NIH, USA).

### TUNEL assay

TUNEL assay was carried out using paraffin-embedded brain slices prepared from samples harvested at 48 h after Sham or TBI operation as previously described [60] with the TUNEL detection Kit (C1088; Beyotime Biotechnology, Shanghai, China). In brief, after washing twice in PBS, 4 μm thick brain slices were dewaxed, re-hydrated, and treated with Proteinase K (10 μg/mL, pH 7.5–8.0, 15 min at 37°C). After rinsing with PBS, the tissue sections were stained and the nuclei were stained with DAPI (Vector Laboratories, Inc., USA). The stained tissue slices were then mounted and imaged under a fluorescence microscope (Olympus Corporation, Tokyo, Japan). Total count of neurons and the numbers of TUNEL-positive neurons were determined in five fields that were randomly selected under the microscope at 200x magnification. The percentage of apoptotic neurons were calculated as (TUNEL-positive neurons/total neurons) × 100%.

### Immunofluorescence

Immunofluorescence staining was performed with 12-μm thick frozen brain tissue slices. The sections were processed for 1 h at room temperature in 10% normal goat serum (ab7481; Abcam). Then, the samples were incubated with primary antibodies against Nrf2 (ab137550; 1:200; Abcam) or NeuN (94403; 1:100; Cell Signaling) at 4°C overnight, and then labeled with an Alexa Fluor^®^ 488 goat anti-rabbit IgG secondary antibody (ab150077; 1:1,000; Abcam) or Alexa Fluor^®^ 647 goat anti-mouse IgG (H + L) secondary antibody for 1 h at room temperature. DAPI was used for nuclear staining in immunofluorescence staining. The images were photographed using a confocal laser-scanning microscope (F1000, Olympus, Tokyo, Japan) and fluorescence data were processed by using the MATLAB software (MathWorks, USA).

### Statistical analyses

SPSS version 23 (SPSS, USA) was used for data analysis and shown as mean ± SD or mean ± SE as indicated. All experiments were implemented in triplicates. For the behavioral analysis and mNSS, a two-way mixed-model analysis of variance (ANOVA) and Sidak’s post-hoc test were performed. Additional data were compared between multiple groups by one-way ANOVA with Tukey’s post-hoc test. Two-sided *P* < 0.05 was considered statistically significant.
